# γ-Tocotrienol Induces Paraptosis-Like Cell Death in Human Colon Carcinoma SW620 Cells

**DOI:** 10.1371/journal.pone.0057779

**Published:** 2013-02-28

**Authors:** Jing-Shu Zhang, Da-Ming Li, Yue Ma, Ning He, Qing Gu, Feng-Shan Wang, Shu-Qing Jiang, Bing-Qing Chen, Jia-Ren Liu

**Affiliations:** 1 Department of Toxicology, Tianjin Center for Disease Control and Prevention, Tianjin, People’s Republic of China; 2 College of Public Health, Tianjin Medical University, Tianjin, People’s Republic of China; 3 Department of Nutrition and Food Hygiene, Public Health College, Harbin Medical University, Harbin, People’s Republic of China; 4 Harvard Medical School (CHB), Boston, Massachusetts, United States of America; National Cancer Center, Japan

## Abstract

Colorectal cancer is one of the most serious illnesses among diagnosed cancer. As a new type of anti-cancer composition from tocotrienol-rich fraction of palm oil, γ-tocotrienol is widely used in anti-cancer research. The objectives of this study were to investigate the effects of γ-tocotrienol on human colon cancer SW620 and HCT-8 cells. We showed that treatment with different concentrations of γ-tocotrienol resulted in a dose dependent inhibition of cell growth. Cell death induced by γ-tocotrienol was mediated by a paraptosis-like cell death in SW620 and HCT-8 cells. Real-time RT-PCR and western blot analyses showed that γ-tocotrienol inhibited the expression level of β-catenin, cyclin D1 and c-jun. These data suggest that a paraptosis-like cell death induced by γ-tocotrienol in SW620 cells is associated with the suppression of the Wnt signaling pathway, which offers a novel tool for treating apoptosis-resistance colon cancer.

## Introduction

Cancer is serious problems in public health, and has become one of the main death causes all around the world. Among diagnosed cancer, colorectal cancer is the third most common type of cancer in men and the second in women worldwide, with 663,600 in men, 570,100 in women new colorectal cancer cases and 320,600 in men, and 288,100 in women estimated deaths occurred in 2008 [1]. The reasons that cause this kind of state are varied, including overweight and obesity, physical inactivity, changes in dietary patterns, and an increased prevalence of smoking [2]. Most patients with colon cancer have accompanied with the activation of the Wnt/β-catenin signal pathway [3], [4]. In a study [5], high activities of Wnt/β-catenin signaling pathway could be one of the mechanisms that drive the colitis-to-cancer transition in human colon.

Using the anti-cancer components in natural products to intervene tumor occurrence and stop cancer from developing, which is the filed for nutritionists to explore actively. One of characteristic features of chemotherapeutic agents from natural products is specific killing of malignant cancer cells but little toxicity to normal cells. Some anti-cancer components, like conjugated linoleic acid [6], [7], β-ionone [8], [9], curcumin [10], berberine [11] are available from natural products. Tocotrienols, a new potential natural chemotherapeutic agent, are abundant in rye, barely, oat and palm oil [12]. Tocotrienols have been reported to have a variety of therapeutic functions, including anti-oxidant activity, anti-cancer activity, anti-angiogenic and immunomodulatory effects [13]. Tocotrienols, as a fat-soluble isomer of vitamin E, absorbed in a similar fashion as fat from food in the intestines. Their peaks in the blood are about 4 h after ingestion, and their absorption is known to absorb more readily when taken along with meals [14]. It is known that tocotrienols are one of kinds of vitamins which are necessary in human being, rather than a kind of pharmaceutical drugs. In a clinical trial study [15], tocotrienols have the immunomodulatory effects in healthy women who taken 400 mg TRF-supplement (tocotrienol-rich fraction from palm oil) daily when compared to the placebo group. A significantly increasing plasma level of total vitamin E was found in the TRF-supplemented group. Tocotrienols contain α-, β-, γ-, and δ-homologues, with different number of methyl groups, and their biological activities are also different, for example, γ-tocotrienol is more active than δ-tocotrienol in Hela cells [16], while the anti-cancer capacity of tocotrienols in prostate cancer cell lines is δ-tocotrienol>γ-tocotrienol>β-tocotrienol>α-tocotrienol [17]. Tocotrienols also may modulate various molecular targets, such as 3-hydroxy-3-methyl-glutarylcoenzyme A (HMG-CoA) reductase, inﬂammatory transcription factors, and death receptors, at the transcriptional, translational, post-translational levels [13]. γ-Tocotrienol also induced apoptosis in human gastric adenocarcinoma SGC-7901 cells and human colon carcinoma HT-29 cells, which is associated with suppression of the Raf-ERK signaling pathway [18] and mitogen-activated protein kinase signaling pathway [19], inhibitory effects on cell invasion and metastasis [20], [21]. However, the exact molecular mechanisms for cell death induced by tocotrienols are still unclear.

Although many studies show that tocotrienols can induce apoptosis in many kinds of malignant carcinoma cells by a cancer-killing activity, tocotrienols also induce a programmed non-apoptosis cell death through caspase-independent programmed cell death (CI-PCD) in several types of cancer cells. Our previous results have also demonstrated that δ-tocotrienol induced a paraptosis-like cell death in human colon carcinoma SW620 cells [22].

Paraptosis, a new type of CI-PCD, is characterized mainly by a process of cytoplasmic vacuolization [23]. Typical apoptotic morphology, such as pyknosis, caspase activation and DNA fragmentation, is absent in this form of cell death [24]. Vacuolation has been recognized as the results from swelling of mitochondria and the endoplasmic reticulum (ER). AIP1/Alix, a protein cloned from a calcium-binding protein involved in T-cell receptor induced cell death, is the specific inhibitor for paraptosis induced [24], which means that paraptosis requires protein synthesis and transcription. Although paraptosis and paraptosis-like processes have been described in various cell models, the exact mechanisms underlying paraptosis are also still unclear.

PTX (paclitaxel), a kind of natural plant extract, is applied to the cancer clinical treatments. PTX also shows induction of apoptosis on several kinds of cancer cells such as colon cancer HT-29 cell [25] and pancreatic cancer cells [26]. pone.0044418-Handeli1In this study, we use the PTX as a negative control to exam the effects of γ-tocotrienol on cytoplasmic vacuolization. In addition, in order to explain the relationship between the paraptosis-like deaths in SW620 cells, FH535 which is an inhibitor of the Wnt pathway was used in this study.

In this study, we investigated the effects of γ-tocotrienol in SW620 cells. In order to rule out the cell line-based bias, we choose another colon cancer cells (HCT-8 cells) carry out some parallel experiments. We observed the cytotoxicity and the morphological changes when treated with γ-tocotrienol. The result showed that γ-tocotrienol induced a paraptosis-like cell death in SW620 cells characterized by a mass of cytoplasmic vacuoles formation, which may associate with the suppression of the Wnt signaling pathway.

## Results

### γ-Tocotrienol Inhibited Cell Proliferation of SW620 Cells

**Figure 1:**
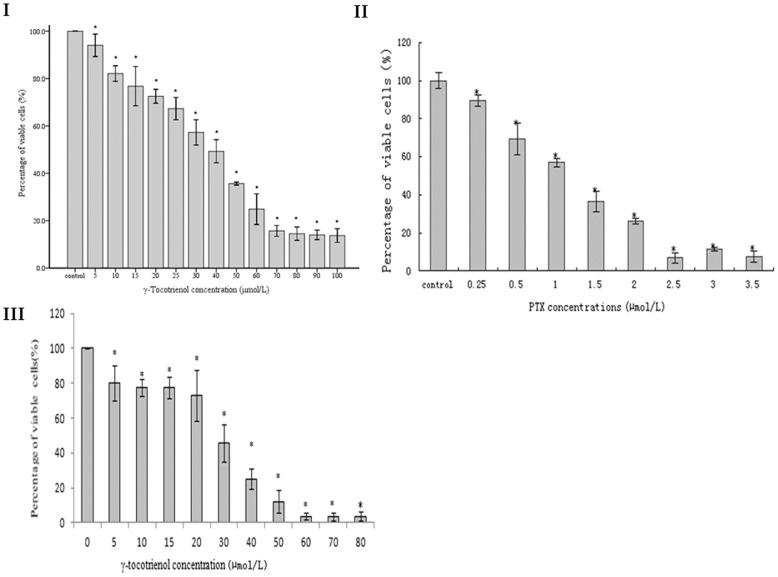
The cell viability of SW620 cells and HCT-8 cells. The panels showed γ-tocotrienol (I) and PTX (II) in SW620 cells and HCT-8 cells (III). The cells were treated with various concentrations of γ-tocotrienol or PTX for 24 h. Cell viability was determined by MTT assay. Data were expressed as means ± standard deviation (n = 6). *P<0.05, compared to the negative control group.

The effect of γ-tocotrienol on SW620 and HCF-8 cell proliferation was examined by MTT assay. As shown in Fig. 1, γ-tocotrienol significantly inhibited cell growth in SW620 and HCF-8 cells in a dose-dependent manner (p<0.05). The inhibitory rates of γ-tocotrienol on SW620 cells for 24 h were 77.5% at dose of 60 µmol/L when compared to the control group. The IC_50_ value of γ-tocotrienol was 31.4±1.51 µmol/L in SW620 cells and 32.69±1.29 µmol/L in HCT-8 cells. In the meantime, the cell viability of PTX in SW620 cells also was determined in this study. The results showed that PTX significantly inhibited cell growth in SW620 cells in a dose-dependent manner (p<0.05).

### γ-Tocotrienol Induced Paraptosis-like Cell Death

**Figure 2:**
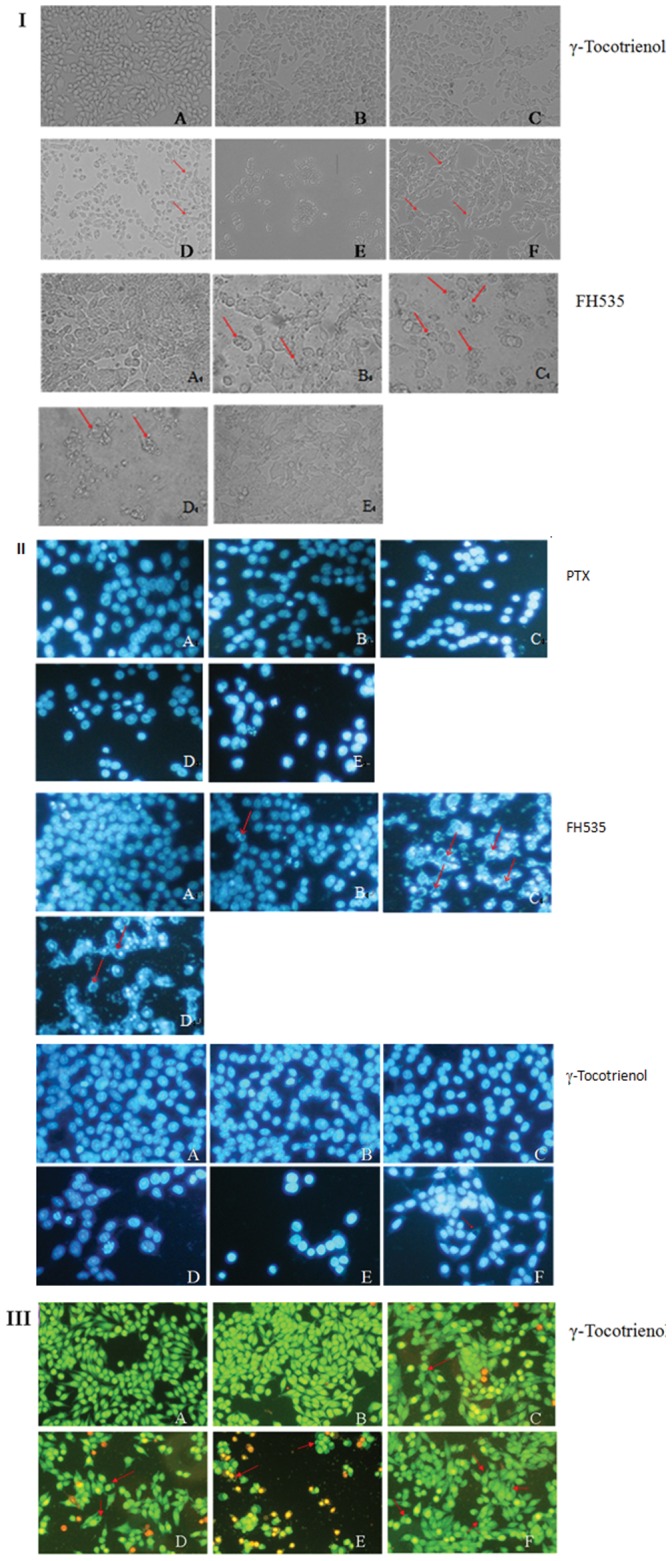
The morphological changes of cell death in SW620 cells treated with γ-tocotrienol, FH535 or PTX. SW620 cells were treated with γ-tocotrienol, FH535 or PTX for 6 and 24 h. The morphological changes of SW620 cells were detected under a light microcopy (I, Magnification: ×100), a fluorescent microscope stained with DAPI (II, magnification: ×200) and AO/EB (III, magnification: ×100). The cells treated with control (A), 15 (B), 30 (C), 45 (D) and 60 µmol/L (E) for 24 h as well as 60 µmol/L of γ-tocotrienol (F) for 6 h, or the cells treated with PTX at doses of 0(A), 0.2(B), 0.4(C), 0.8(D), 1.2(E) µmol/L, or FH535 at doses of 0(A), 0.625(B), 1.25(C), 2.5(D) µmol/L and DMSO (E) for 24 h. After treatment, the cells showed the slow growth, loosed tight contacts amongst cells and reduction of cell size, turn round of cell shape and cytoplasmic vacuolar when compare a spindly and large and grew close together, with prominent nucleoli and abundant cytoplasm in control group. Red arrows indicate the vacuoles.

**Figure 3:**
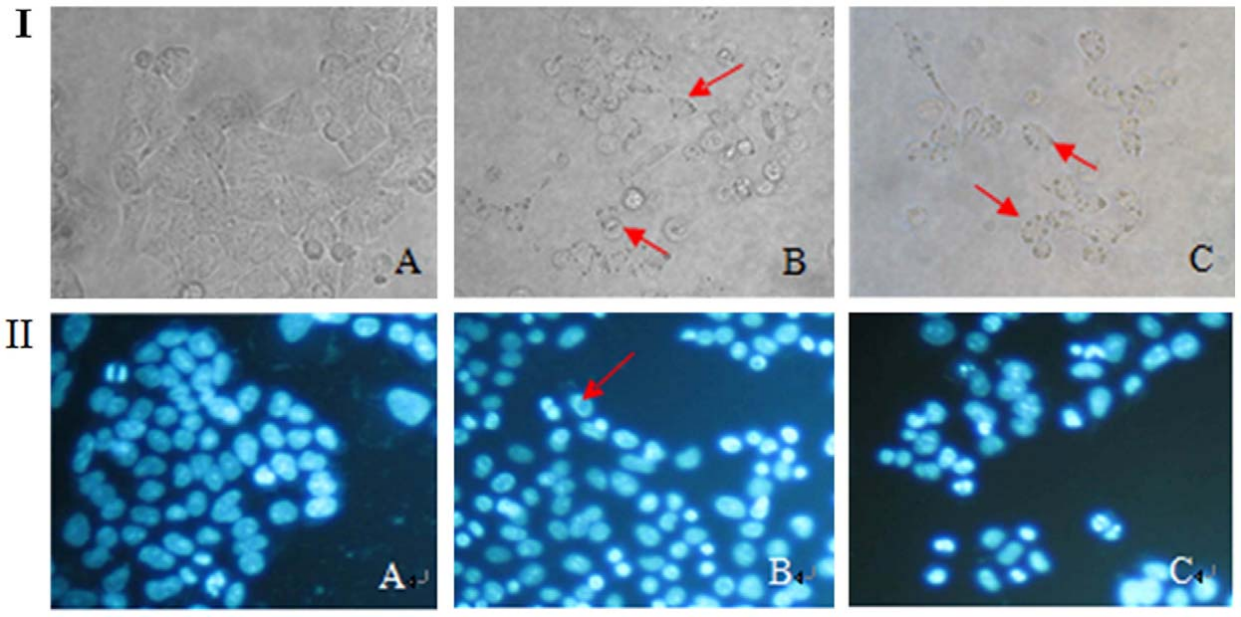
The morphological changes of cell death in HCT-8 cells. HCT-8 cells were treated with 0, 20, and 40 µmol/L of γ-tocotrienol for 24 h. The morphological changes of HCT-8 cells were detected under a light microcopy (I, Magnification: ×200). The cells were stained with DAPI (II, Magnification: ×200). Both figure showed that the cells treated with control (A), 20 (B), 40 µmol/L(C) for 24 h. Arrows indicate the vacuoles.

Cell death was further investigated in SW620 and HCT-8 cells in this study. The results are shown in Fig. 2 and 3. Microscopic findings showed that the cells showed a spindly and large and grew close together, with prominent nucleoli and abundant cytoplasm in control group and the treated cells grew slowly and loosed tight contacts amongst cells (Fig. 2 and 3I). In addition, we also observed reduction of cell size, turn round of cell shape and cytoplasmic vacuolar in treated SW620 cells. The cells treated with γ-tocotrienol at dose of 60 µmol/L for 6 and 24 h showed a cytoplasmic vacuolization and the dead cells with extensive cytoplasmic vacuolization, respectively. The same phenomenon of cellular morphological changes also was observed in FH535 treated groups (Fig. 2I).

To better understand cellular morphological changes induced by γ-tocotrienol, SW620 and HCF-8 cells were analyzed by staining DAPI or AO/EB dyes or transmission electron microcopy (TEM). As shown in Fig. 2 and 3 II, the cells were no obvious changes on cell nucleus accompanying with increasing concentration of γ-tocotrienol. After were exposed to γ-tocotrienol for 6 h, SW620 cells showed an intracellular vacuolization appeared on the black background (Fig. 2 II F). Interestingly HCT-8 cells also showed an intracellular vacuolization appeared on the black background when treated with γ-tocotrienol (Fig. 3 II B). The results of AO/EB staining are shown in Fig. 2 III. The treated cells did not show a typical apoptosis such as apoptosis body and chromatin condensation. After γ-tocotrienol treatment at dose of 60 µmol/L for 6 and 24 h, cells showed numerous vacuoles (Fig. 2 III F) and high degree of cell rounding and large vacuoles (Fig. 2 III E), respectively. The same phenomenon of cellular morphological changes also was observed in the FH535 treated group. However, they did find evident cellular morphological changes in the PTX treated group when compared to the FH535 group (Fig. 2II).

**Figure 4:**
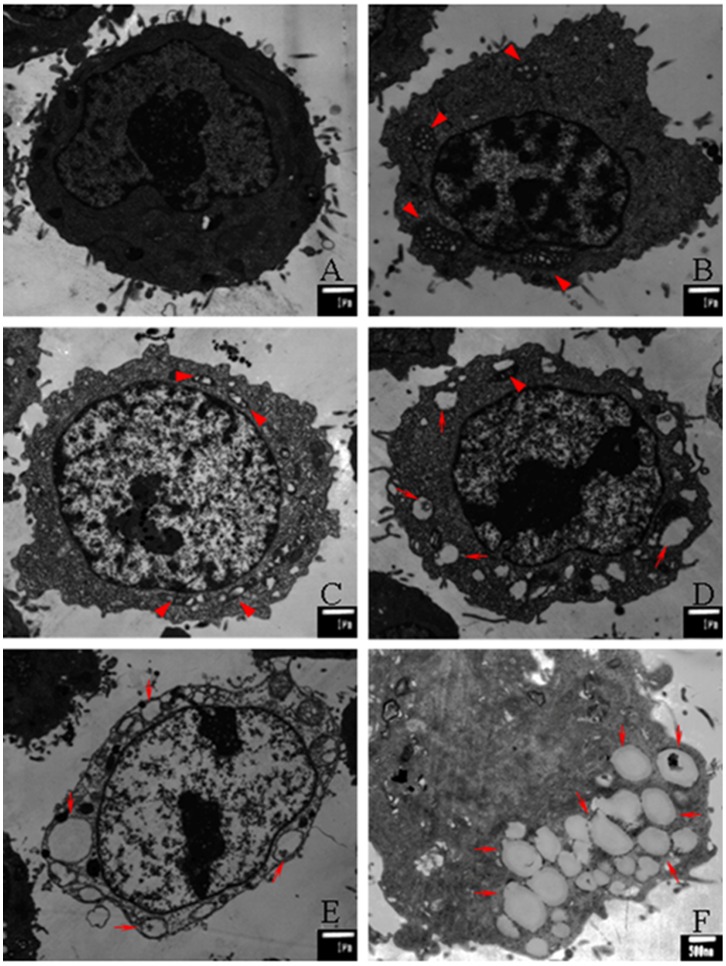
Morphological changes of paraptosis-like cell death were observed in SW620 cells by transmission electron microscopy (TEM). After treatment with 15 (B), 30 (C), 45 (D) and 60 µmol/L (E) of γ-tocotrienol for 24 h, SW620 cells showed the swelling of mitochondria or ER (red arrowheads) and extensive cytosolic vacuolization (red arrows). The micrographs (A) provided normal cells control. The significant swellings of mitochondria or ER were observed after treated with 60 µmol/L of γ-tocotrienol for 6 h (F).

In order to detect the γ-tocotrienol-induced vacuoles in SW620 cells, the transmission electron microscopy (TEM) was emplyeed to observe the cellular ultrastructures. The results showed that swelling of the mitochondria and/or endoplasmic reticulum (ER) was detected in the cells treated with γ-tocotrienol (Fig. 4). The characteristics of classic apoptosis did not found in this study. The cells showed intact and clear organs in the control group. After treatment for 24 h, swell and formed megamitochondria were observed in the cells treated with 15 or 30 µmol/L of γ-tocotrienol groups (Fig. 4 B and C). The rates of fusion of megamitochondria and/or ER decreased and the number of vacuoles increased in the cells treated 45 and 60 µmol/L of γ-tocotrienol groups (Fig. 4D). In addition, after treatment 6 h, a plenty of large or small cytoplasmic vacuoles were observed in the cells treated with 60 µmol/L of γ-tocotrienol (Fig. 4 F). Taken together, these results demonstrate that γ-tocotrienol induces paraptosis-like cell death in SW620 cells.

### Determination of Cell Growth State Distribution and Activated Caspase-3

**Figure 5:**
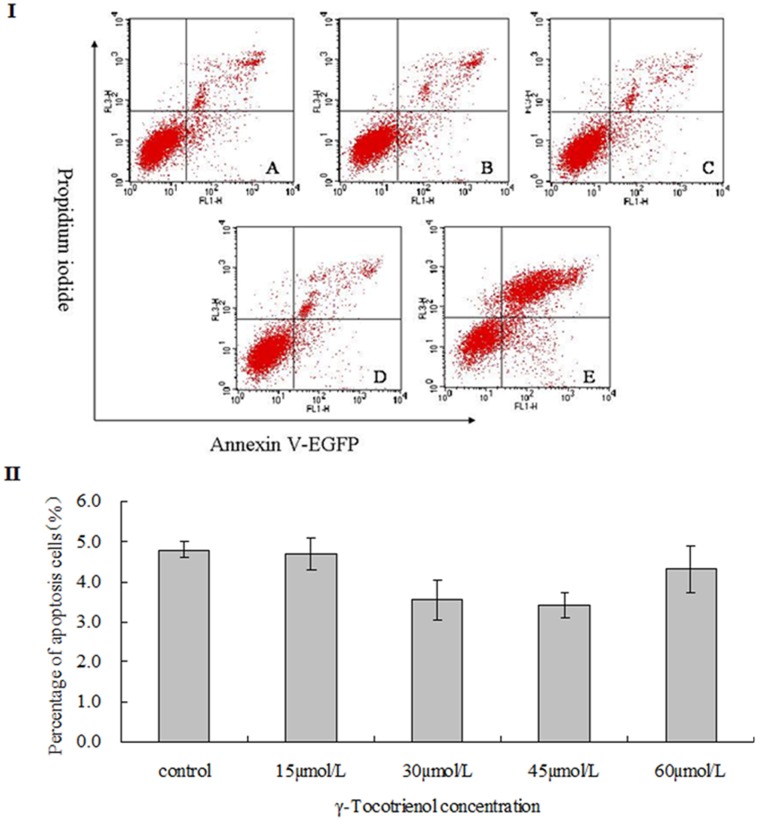
Cell death was examined in SW620 cells by flow cytometry. The cells treated with 0 (A), 15 (B), 30 (C), 45 (D) and 60 µmol/L (E) of γ-tocotrienol for 24 h. The percentage of early apoptosis was obtained from lower right quadrant (annexin V-EGFP^+^, PI^–^). *P<0.05, compared to the same dose of each group.

**Figure 6:**
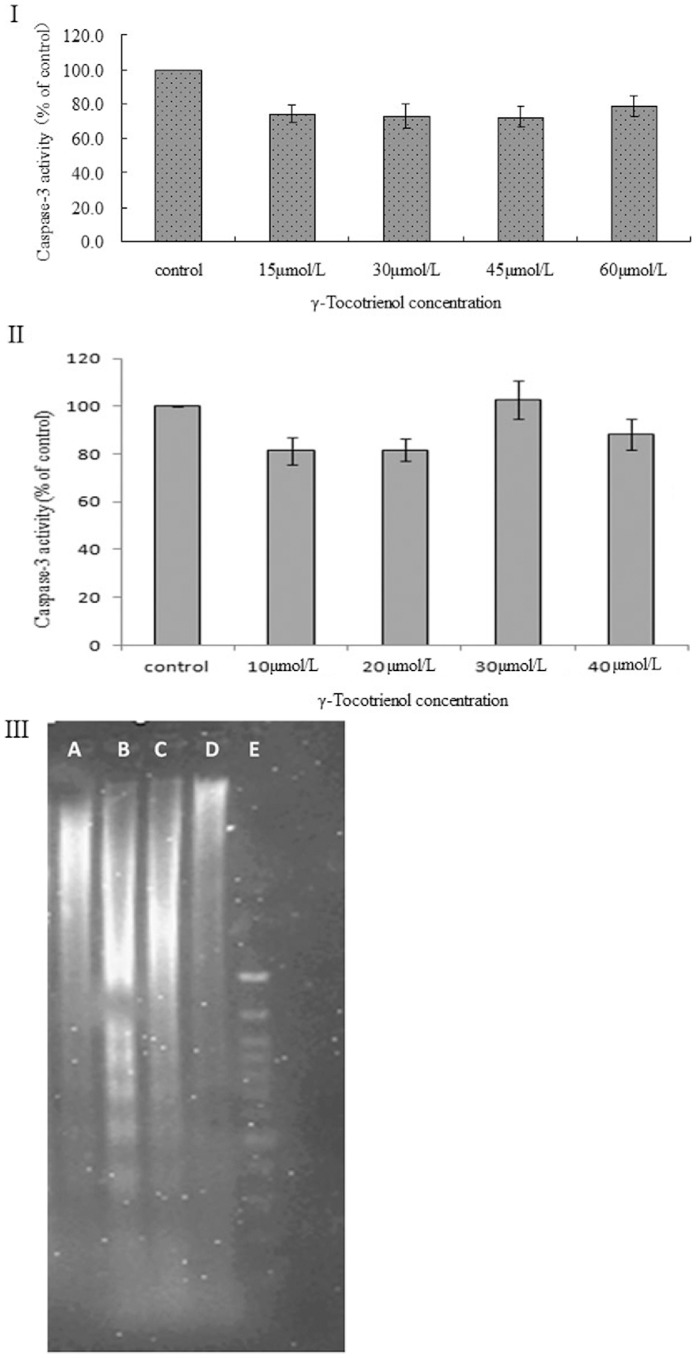
The cellular caspase-3 activity and DNA ladder were examined in SW620 cells or HCT-8 cells. The cellular caspase-3 activity was determined in SW620 cells (I) and HCT-8 cells (II) treated with different doses of γ-tocotrienol as well as DNA ladder in SW620 cells treated with PBS (A), 1.0 and 1.5 µmol/L of PTX or γ-tocotrienol (D) for 24 h. There are no difference between the treated and control groups.

**Figure 7:**
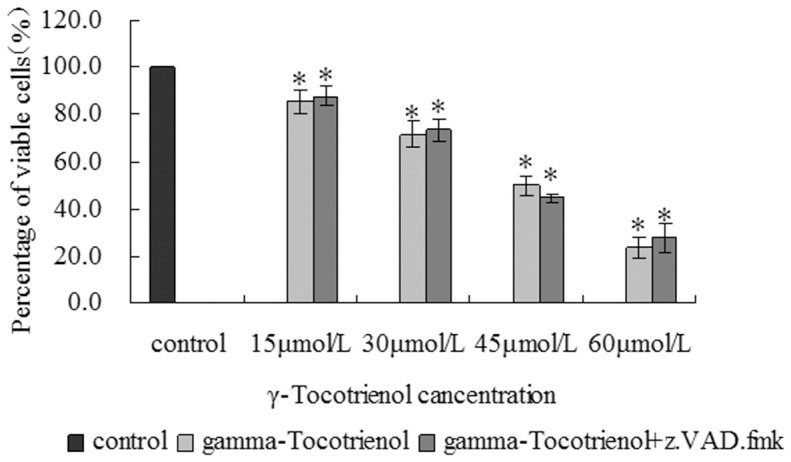
The cell viability of γ-tocotrienol with or without z.VAD.fmk. The SW620 cells were treated with different doses of 15, 30, 45 and 60 µmol/L of γ-tocotrienol with or without z.VAD.fmk for 24 h. The cell viability was examined by MTT assay. *P<0.05, compared to the control group.

We further examined the cell growth state distribution and activation of caspase-3 in SW620 cells treated with γ-tocotrienol for 24 h. The cell growth state distribution was determined by flow cytometry. By using the Annexin V-EGFP and PI dye, SW620 cells were divided into three types including viable cells (annexin V-EGFP^–^, PI^–^), non-viable necrotic cells in late apoptotic cells (annexin V-EGFP^+^, PI^+^) and early apoptotic cells (annexin V-EGFP^+^, PI^–^). As shown in Fig. 5 and Fig. 6, the proportion of early apoptotic cells was below 6.0% in all groups, and a peak of cell death was found in SW620 cells treated with 60 µmol/L of γ-tocotrienol. In order to determine caspase-3 activation, SW620 cells were pre-treated with z.VAD.fmk, a pan-specific caspase inhibitor and then treated with different concentrations of γ-tocotrienol for 24 h. The rate of cell viability was measured in the treated and control groups (Fig. 7). The results showed that the percentage of viable cells was declined in all groups, and γ-tocotrienol had the same effect on SW620 with/without z.VAD.fmk. It indicated that γ-tocotrienol-induced cell death is caspase-independent. Caspase-3 activation was also examined in this study. The relative ratios of caspase-3 activation were 74.3±5.2, 72.9±7.3, 72.4±6.3 and 78.6±5.8% in SW620 cells treated with 15, 30, 45 and 60 µmol/L of γ-tocotrienol, respectively (Fig. 6 I). There was no significant change among the treated groups and the control group (*P*>0.05). As shown in Fig. 6 II, the caspase-3 activity also determined in HCT-8 cells after treated with different doses of γ-tocotrienol (10, 20, 30 and 40 µmol/L). The results showed that γ-tocotrienol-induced cell death in HCT-8 cells was also caspase-independent. In the meantime, DNA ladder assay also used to determine apoptosis in this study. The results showed that PTX at doses of 1.0 and 1.5 µmol/L induced the formation of DNA ladder in SW620 cells. γ-Tocotrienol did not induce the formation of DNA ladder in SW620 cells (Fig. 6 III).

### γ-Tocotrienol Affected the Protein Expression of Wnt Signaling Pathway

**Figure 8:**
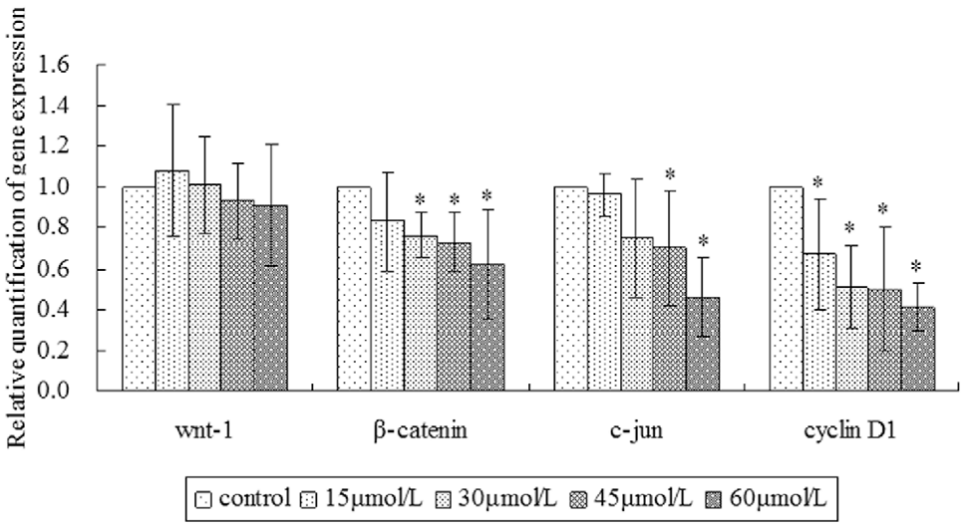
The mRNA expression levels of Wnt-1, β-catenin, c-jun and cyclin D1 were detected in SW620 cells treated with different concentration of γ-tocotrienol by RT-PCR. γ-Tocotrienol significantly decreased the mRNA expression of β-catenin, c-jun and cyclin D1 in SW620 cells (*P*<0.05).

Own to Wnt signaling pathway playing a critical role in tumor occurrence and development, the effects of γ-tocotrienol was investigated in SW620 cells by real-time PCR and western blot. The mRNA expression levels of Wnt-1, β-catenin, c-jun and cyclin D1 are shown in Fig. 8. β-Catenin, c-jun and cyclin D1 expression showed a decrease accompanying with the increasing concentrations of γ-tocotrienol. The mRNA levels of c-jun and cyclin D1 were significantly decreased in SW620 cells treated with 45 or 60 µmol/L of γ-tocotrienol when compared to the control group (*P*<0.05). Although the mRNA expression of Wnt-1 at dose of 15 µmol/L group was slightly higher than that in the control group, there was no differences (*P*>0.05). This result indicated that γ-tocotrienol had no effect on the mRNA expression of Wnt-1.

**Figure 9:**
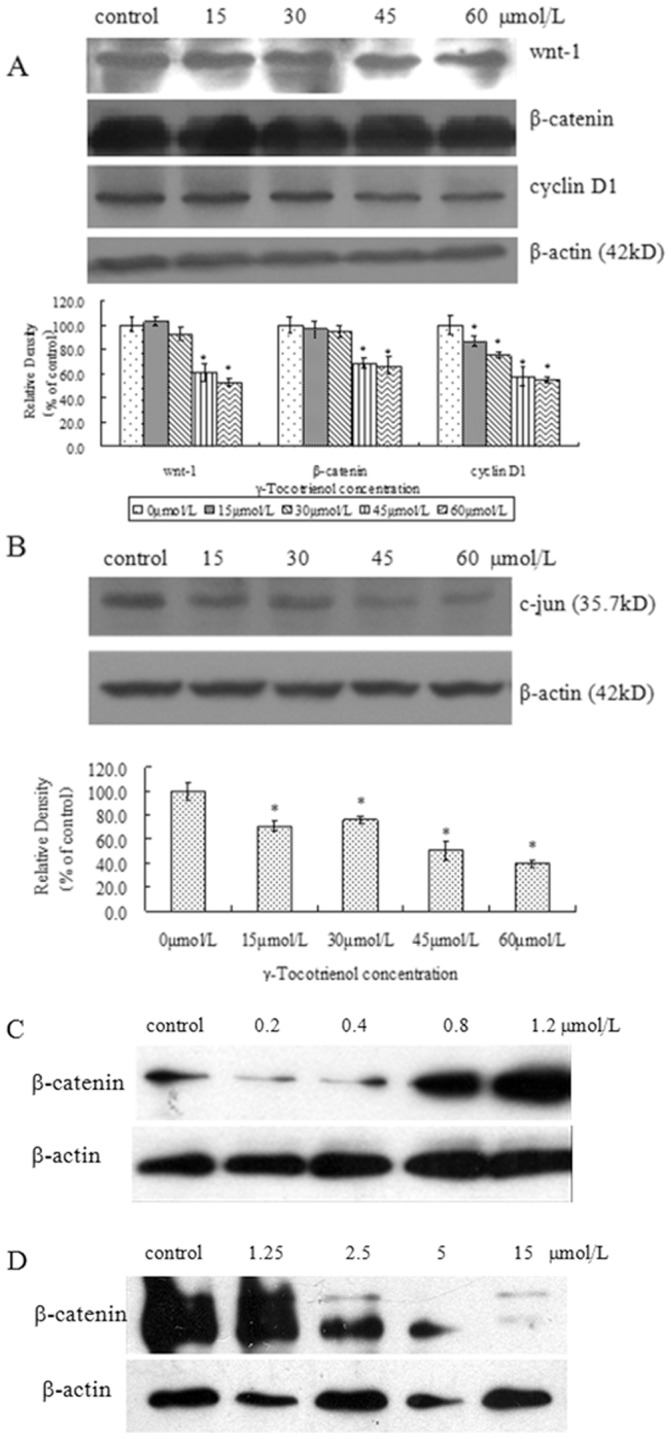
The protein expressions of Wnt-1, β-catenin, c-jun, cyclin D1 and β-actin in SW620 cells. The cells treated with 15, 30, 45 and 60 µmol/L of γ-tocotrienol (A and B), or PTX at 0.2, 0.4, 0.8 and 1.2 µmol/L (C), or FH535 at 1.25, 2.5, 5 and 15 µmol/L for 24 h (D). *P<0.05, compared to the negative control group.

We further examined whether protein expression associated with Wnt signaling pathway were modulated by γ-tocotrienol. As shown in Fig. 9, γ-tocotrienol significantly down-regulated the protein expression of Wnt-1 and β-catenin as well as the downstream target proteins of Wnt signaling pathway such as c-jun and cyclin D1 in a dose-dependent manner (p<0.05 or p<0.01). In addition, PTX at doses of 0.8 and 1.2 µmol/L significantly increased β-catenin protein expression and FH535 significantly decreased β-catenin protein expression in SW620 cells.

## Discussion

Anti-cancer agents found in natural products have been reported to preferential killing of cancer cells [27]. As one of the most important components of vitamin E, tocotrienols are the main bioactive components in palm oil, which not only has higher antioxidant potential but also more other bioactivity than tocopherol, especially in the potent ability on killing cancer cells [28]. Many studies have been reported tocotrienols and tocotrienol-rich fraction from palm oil (TRF) can kill several kinds of malignant carcinoma cells inhibit the growth of many kinds of tumors in animals [29]–[31]. TRF can not only inhibit tumor growth by reducing revascularization [29], but also affect tumor gene expression in the nude mouse body inoculated by MCF-7 cells [30]. TRF also showed an evident up-regulation of the interferon-inducible transmembrane protein-1 gene and the CD59 glycoprotein precursor gene in TRF-treated animals in comparison with controls [30]. Another study showed that γ-tocotrienol could inhibit SGC-7901 cell growth and increase the chemosensitization of capecitabine in a xenograft mouse model through the modulation of NF-κB pathway [31].

Tocotrienols can induce different types of cancer cell death. A series of studies show that tocotrienols induce apoptosis in many cancer cells, such as MDA-MB 231 and MCF-7 breast cancer cells [32], PC-3 and LNCaP prostate cancer cells [33] and non-small cell lung cancer cells [34]. In a previous study, γ- and δ-tocotrienols induced CI-PCD in prostate cancer cell lines by inactivation of caspase-3/8 [17] and caspase-independent DNA damage [35]. Several evidences show that tocotrienols can induce autophagy [33], [36], [37]. γ-Tocotrienol induced autophagy in prostate cancer cells by elevation of intracellular dihydroceramide and dihydrosphingosine [33]. In another study, tocotrienols induced autophagy in rat pancreatic stellate cells through the mitochondrial death pathway [37]. In addition, γ-tocotrienol also induced typical morphological characteristics of apoptosis in MDA-MB-231 human breast cancer cells, with no change of bax and bcl-2 expression and absence of poly-(ADP-ribose)-polymerase cleavage [36]. Therefore, these studies indicated that an alternative CI-PCD may be involved in the cell death induced by tocotrienols.

Tocotrienols have α-, β-, γ- and δ-tocotrienol isomers. Previous studies have shown that the isoforms of tocotrienols have different biological activities. The anti-cancer capacity of these isomers on human hepatocellular carcinoma HepG2 cells is δ-tocotrienol>β-tocotrienol>α-tocotrienol ≈ γ-tocotrienol [38]. δ-Tocotrienol has a higher activity than other tocotrienols on reducing fibroblast growth factor-induced proliferation, migration, and tube formation in HUVEC [39]. Our previous study showed that δ-tocotrienol inhibited cell proliferation of SW620, and the IC50 value was 15.2 µmol/L [22]. γ-Tocotrienol and α-tocotrienol are more potent than δ-tocotrienol on the antiproliferative effect on human cervical carcinoma (Hela) cells, with the IC50∶2.85±0.07 µmol/L, 3.19±0.05 µmol/L and >100 µmol/L, respectively [16]. Another study showed that γ-tocotrienol is more active than α-tocotrienol and δ-tocotrienol on inducing apoptosis in human hematological cancer cell lines [40]. In our previous study, γ-tocotrienol inhibited cell proliferation in human colon carcinoma HT-29 cells (IC50∶31.7 µmol/L) [41] and human gastric adenoma carcinoma SGC-7901 cells (IC50∶61.88 µmol/L) [42]. Compared with IC 50 dosage of induction cell death of tocotrienols in human colon carcinoma SW620 cell, γ-tocotrienol is larger than that in δ-tocotrienol. Thus, γ-tocotrienol had a weak cytotoxic effect on SW620 cells in comparison with δ-tocotrienol’s. It may be the reason to explain why γ-tocotrienol and δ-tocotrienol are different because of their different stereochemical structure. In addition, tocotrienols at micromole dosage inhibit the proliferation of cancer cells and no cytotoxicity in normal cells [43]. In a previous study, δ-tocotrienol induced either no or low levels of apoptosis in normal human mammary epithelial cells and immortalized but no tumorigenicity in human MCF-10A cells [43]. γ-Tocotrienol showed no cytotoxicity on normal human peripheral blood mononuclear cells, suggesting that tocotrienols have a differential cytotoxicity towards normal lymphocytes and transformed lymphoma cells [44].

Classical apoptosis is caspase-dependent programmed cell death (CD-PCD). While accumulating evidence in the literature supports that several types of caspase-independent programmed cell death (CI-PCD) exist in the cells, such as autophagy, cornification, mitotic catastrophe, anoikis, paraptosis, excitotoxicity, Wallerian degeneration and programmed necrosis [45]. Therefore, it may be a new way for treating malignant cells to understand the exact mechanism on the different types of CI-PCD. In our study, γ-tocotrienol induced vacuolation before cell death in SW620 and HCF-8 cells, with no characteristics of apoptosis, such as cellular shrinkage, apoptotic bodies and activation of caspase-3. Moreover, TEM analysis revealed that vacuolation in SW620 cells came from the swelling and subsequent fusion of mitochondria or ER. In addition, the pan-caspases inhibitor z.VAD.fmk had no effect on γ-tocotrienol-induced cell death and the rates of early apoptosis in γ-tocotrienol treated were similar to that of the control group. These suggest that this kind of cell death induced by γ-tocotrienol is different from apoptosis.

Because of the instability of cancer cells, the mutations are often occurred, leading resistance of cancer cells to current anti-apoptosis therapies. Therefore, CI-PCD may play an important role in inducing cell death and be the major death style in many tumor cells [46]. Many natural anti-cancer substances from food such as curcumin [47], β-ionone [48], conjugated linoleic acid [49], taxol [50], ginsenoside Rh2 [51], as well as other chemotherapeutic agents [52] like doxorubicin, paclitaxel, can induce CD-PCD and CI-PCD in cancer cells.

The cross-talk between cell death signaling pathways may play an important role in switching cell death among the death programs. It is reported that more than one kind of cell death program may be activated at the same time in the same cells [53]. The dominant cell death phenotype is determined by the fastest and most effective pathway on the available death programs [52]. According to this, paraptosis is the dominant cell death in γ-tocotrienol-induced cell death in SW620 cells. In addition, organelles such as the mitochondria and ER are reported to play a prominent role in paraptosis [24], [51], [54]. In our study, our results showed that γ-tocotrienol induced a paraptosis-like cell death in human colon carcinoma SW620 cells. Furthermore, we further determined the possible molecular mechanism of cell death induced by γ-tocotrienol in human colon cancer SW620 cells.

The Wnt signaling pathway plays very important roles in stem cell maintenance, cell proliferation and differentiation regulation [55]. It is also known that the Wnt signaling pathway participates in tumorigenesis in many human cancers, including colon cancer, breast cancer, lung cancer, melanomas, and hepatocellular carcinoma[56]–[61]. The Wnt signaling pathway is activated in approximately 90% of human colorectal cancer [4] and crucial for colon cancer stem cells [62]. The level of β-catenin controls the canonical pathway and is regulated by the destruction complex, consisting of two ser/thr kinases, GSK-3β (glycogen synthase kinase-3β) and CKI, two scaffold proteins, Axin and APC (adenomatous polyposis coli) [63]. When the Wnt/wg ligand is absent, Axin complex in the cytoplasm showing a stable state, CK1α, GSK-3β in this complex sequentially phosphorylated β-Catenin. Phosphorylated β-Catenin could be recognized by the E3 ubiquitin ligase enzyme β-Trcp, and then be de-gradated by proteasome, to ensure the concentrate of the β-catenin maintained at a relatively low level [58]. Once Wnt signaling is activated, Axin complex depolymerization, β-catenin in cytoplasm has the chance of cumulate, and then combine with the TCF (T-cell factor) in cell nuclear to activate the Wnt gene. The activated Wnt genes result in inducing the downstream genes (such as c-jun and c-myc) transcription which are associated with malignancy, and ultimate affect the activities of cell [64]. The Wnt antagonists are divided into two categories: one direct bind to Wnt to inhibit the Wnt signaling, including secreted frizzled-related proteins (SFRPs), Wif-1 and Cerberus; the other combines writ receptor composite LRPS/LRP6 to inhibit the Wnt signaling pathway. Especially, SFRPs were admitted as a family of new colorectal tumor suppressor candidate genes [65]. SFRP-1 and the SFRP-like molecule V3Nter can inhibit Wnt signaling and expression of the β-catenin target genes - cyclin D1 and c-myc, finally inhibiting tumor growth of β-catenin-activated tumor cells such as colon cancer SW480 and HCT-116 cells [66]. Therefore, the Wnt/β-catenin signaling pathway is an attractive target for new therapeutics. A series of our previous studies showed that tocotrienols mediated grow inhibition on human colon cancer cells that related with suppressing the activity of Wnt signaling pathway [21], [22]. However, there are no further information about the mutation status of the APC gene and β-catenin gene in SW620 cells.

FH535, a kind of cell permeability sulfa compound, could inhibit the Wnt/β-catenin pathway by the function of inhibiting the β-catenin recruitment. In a previous study [67], H535 at the dose of 15 µmol/L could down-regulate β-catenin expression in HCT-116 and SW48 cells. FH535 at the dose of 0.1 µmol/L also showed to inhibit migration and growth of breast cancer cells [68]. Recent studies have demonstrated that FH535 is a synthetic inhibitor of the canonical WNT-signaling pathway and, not for normal fibroblasts, FH535 also inhibits growth of colon, lung, and hepatocellular carcinoma cell lines [67]. In our study, γ-tocotrienol could induce both SW620 and HCT-8 cells death in a type of cytoplasmic vacuolization, and this kind of death did not accord with the typical characters of apoptosis. At the same time the γ-tocotrienol decreased the level of β-catenin in SW620 cells both in protein and mRNA levels. As the target factors, c-jun and cyclin D1 are known to regulate cell growth and vitality and apoptosis [69], γ-tocotrienol also significantly decreased the mRNA and protein expression of c-jun and cyclin D1 in this study. When treated with PTX, the SW620 cells showed the characterics of apoptosis like DNA Ladder. But the level of β-catenin at dose of less 0.8 µmol/L PTX was decreased in SW620 cells. In order to detect the role of Wnt pathway and the relationship between the death of paraptosis in a type of cytoplasmic vacuolization and the Wnt pathway in SW620 cells, a Wnt/β-catenin inhibitor FH535 was used in this study. FH535 could induce SW620 cells death with cytoplasmic vacuolization, and decrease the level of β-catenin protein in the cells. This result indicates that there may be some common ground between γ-tocotrienol and Wnt/β-catenin inhibitor in the mechanism of induce the SW620 cells paraptosis-like death. Bap31 from an endoplasmic reticulum (ER) localized polytopic transmembrane (TM) protein, bax/bax and p20Bap31 (p20) fragment plays a variety of roles in trafficking and quality control paraptosis-like form of cell death [54]. In our previous study, δ-tocotrienol induced paraptosis-like death correlated with the vacuolation that may be from welling and fusion of mitochondria and/or the ER in SW620 cells [22]. Wnt signaling is regulated by ER retention in the mouse embryo [70]. Thus, the paraptosis-like death may be related to Wnt signaling via a regulation of ER. Taken together, our data suggested that cell death induced by γ-tocotrienol in human colon carcinoma SW620 cells is associated with the suppression of the Wnt signaling pathway by a regulation of ER.

## Methods

### Chemicals and Reagents

γ-Tocotrienol (97%) was purchased from Davos Life Science **(**Singapore). 3-(4,5- dimethylthiazol-2-yl)-2,5-diphenyltetrazolium bromide (MTT), ethidium bromide (EB), and acridine Orange (AO) were purchased from Sigma Chemical Co.(St. Louis, MO). 4′, 6-Diamidino-2-phenylindole (DAPI) was obtained from Roche Applied Science (Indianapolis, IN). The caspase inhibitor - benzyloxycarbonyl-Val-Ala-Asp (OMe) ﬂuoromethylketone (z.VAD.fmk), the Caspase-3 Colorimetric Assay kit and Annexin V-EGFP (enhanced green fluorescent protein) Apoptosis Detection kit were bought from Nanjing Keygen Biotech. Co., Ltd. (NanJing, China). Anti-β-actin, anti-Wnt-1, anti-β-catenin, anti-cyclin D1, and anti-c-jun antibodies were purchased from Santa Cruz Biotechnology, Inc. (Santa Cruz, CA). Mouse IgG HRP – and rabbit IgG HRP – secondary antibodies were purchased from Promega (Madison, WI). Real-Time PCR Reagents and Kits were obtained Invitrogen Corporation (Carlsbad, CA). PTX (paclitaxel) and the DNA Ladder Assay Kit were bought from the ZSGB-BIO Company (TianJing, China) and FH535 from Sigma Chemical Co. (St. Louis, MO). All reagents were of analytical grade unless noted.

γ-Tocotrienol was dissolved in 100% ethanol at a concentration of 1.0 mol/L stock solution and stored in dark place at −20°C. The stock solution was diluted to required concentration and stored at 4°C. Experiments were independently carried out at least three times.

### Cell Culture

Human colon carcinoma SW620 and HCF-8 cell lines were obtained from the Cancer Institute of Chinese Academy of Medical Science (Beijing, China) [71], [72]. The SW620 cells were cultured in L-15 medium and HCF-8 cells with DMEM/F12 containing 2 mmol/L L-glutamine, and 2 g/L sodium bicarbonate (Gibco, NY), supplemented with antibiotics (100 U/mL of penicillin and 100 µg/mL of streptomycin), 10% fetal bovine serum (FBS, Gibco) at 37°C in a humidified atmosphere with 95% air and 5% CO_2_. Before subculturing, cells were digested in 0.25% trypsin containing 0.02% EDTA.

### Cytotoxicity Assay

The inhibitory growth of γ-tocotrienol and PTX on SW620 and HCF-8 cells was evaluated according to MTT assay [21], [73]. SW620 or HCF-8 cells (1×10^5^ cells/well) were seeded into 96-well plates. After 24 h, cells were incubated with 200 µL of fresh complete culture medium containing 5, 10, 15, 20, 25, 30, 40, 50, 60, 70, 80, 90 and 100 µmol/L of γ-tocotrienol or 0.25, 0.5, 1.0, 1.5, 2.0, 2.5, 3.0 and 3.5 µmol/L of PTX. The negative control group was treated with 100% ethanol (0.1%v/v). Each concentration of γ-tocotrienol was repeated in six wells. After incubation for 20 h, 20 µL of 1 mg/ml MTT solution in phosphate-buffered saline (PBS) was added to each well. After 4 h, we discarded the medium carefully. Then, 200 µL dimethyl sulfoxide (DMSO) was added to each well. After shaking quickly for 15 sec, the absorbance was measured at weave length of 495 nm in a Multiskan Ascent plate reader (Thermo Labsystems, Helsinki, Finland).

### Morphological Analyses

The SW620 cells were plated in 24-well plates at 5×10^5^ cells/well. After 24 h, the cells were treated with fresh L-15 medium containing 0, 15, 30, 45 and 60 µmol/L of γ-tocotrienol, or 0.625, 1.25, and 2.5 µmol/L of FH535, or 0.2, 0.4, 0.8, and 1.2 µmol/L of PTX. After being treated with γ-tocotrienol for 6 or 24 h, the cells were fixed using methanol. The cells were washed PBS once, treated with DAPI-methanol (1 µg/ml of working solution) and incubated for 15 min at 37°C. The staining solution was then discarded. In the meantime, HCT-8 cells were stained with DAPI solution after γ-tocotrienol treatments. For AO/EB staining assay, after treatment as indicated DAPI staining assay, the cells were washed with PBS twice and stained with AO/EB solution (100 µg/ml of AO and 100 µg/ml of EB in PBS) for 10 sec at room temperature away from light. The stained cells were examined through a fluorescence microscope (IX70, Olympus, Japan).

For transmission electron microscopy analysis (TEM), we have described in previous study [22], [74]. Briefly, after treated with γ-tocotrienol for 6 and 24 h, respectively, the cells were collected, washed with PBS, and fixed in 4% glutaraldehyde phosphate buffer for 24 h at 4°C, followed by 1% osmium tetroxide fixation for 1 h at 4°C and dehydration in graded ethanol solutions. Ultrathin sections counterstained with uranyl acetate and lead citrate were examined under a JEM-1220 TEM (JEMOL, Japan).

### Determination of Cell Death

For the effect of the caspase inhibitor z.VAD.fmk on γ-tocotrienol, the z.VAD.fmk was added to each well at the concentration of 20 µmol/L, with cells continuing to incubate for 10 min. SW620 or HCT-7 cells were then treated with 15, 30, 45 and 60 µmol/L of γ-tocotrienol for 24 h. Cell death was determined as described in Section of cytotoxicity.

For the caspase-3 activity assay, the cells were treated with different concentrations of γ-tocotrienol for 24 h. The activity of caspase-3 was detected according to manufacturer’s instruction.

### DNA Ladder Assay

After PTX treatments, SW620 cells were scraped and collected from culture dishes in ice cold PBS. After centrifugation, the cell pellets were lysed in lysis buffer and centrifuged for 5 min at 1000×g. The supernatant from each sample was collected and DNA was extracted using the assay kit. The DNA samples were electrophoresed in 2% sepharose gel. The DNA bands were analyzed using Gel Documentation and Analysis System.

For FACS analysis, after treatment with various concentrations of γ-tocotrienol for 24 h, 1×10^6^ cells (including the adherent and detached cells) were collected and washed three times with PBS. The cells were resuspended in 500 µL binding buffer, followed by adding 5 µL Annexin V-EGFP and 5 µL propidium iodide (PI) and incubating for 15 min at room temperature. Cell death was performed by a FACSCalibur flow cytometry (Becton and Dickinson, CA) with excitation using a 488 nm argon ion laser.

### Quantitative Real-time RT-PCR Reaction

SW620 cells were treated with 0, 15, 30, 45 and 60 µmol/L of γ-tocotrienol for 24 h. Total RNA from cells were extracted using TRIzol® Reagent (Invitrogen) and first strand cDNA was synthesized from 5 µg of total RNA of each sample utilizing SuperScrip III reverse transcriptase (Invitrogen). The sequences of primers are shown in Table 1.

**Table 1:** The sequences of primers.

Gene	Sequence	Size of PCR product (bp)
Wnt-1	Forward 5′-AGCTACAGGTGGGGAGGAGAA-3′	180
	Reverse 5′-CGGCTCCTGATGGTGTCATT-3′	
β-catenin	Forward 5′-GGCCATATCCACCAGAGTGAA-3′	118
	Reverse 5′-GCCAATGGCTTGGAATGAGA-3′	
cyclin D1	Forward 5′-CTCAGACTTGCGCGTCACAG-3′	196
	Reverse 5′-CAGAACACGGCTCACGCTTA-3′	
c-jun	Forward 5′-TCCCCCAGCTATCTATATGGAAT-3′	148
	Reverse 5′-TCACAGCACATGCCACTTGA-3′	
GAPDH	Forward 5′-AAGAAGGTGGTGAAGCAGGC-3′	203
	Reverse 5′-TCCACCACCCTGTTGCTGTA-3′	

For quantitative real-time PCR, the reactions were performed with SYBR Green I (Invitrogen) according the manufacturer’s instructions. The PCR amplification conditions were as follows: pre-denaturation for 2 min at 95°C; PCR amplification for 40 cycles with denaturation for 10 sec at 95°C, annealing temperature for 30 sec at 60°C, extension for 30 sec at 68°C in a iQ5 real-time PCR System (BIO-RAD). The reaction specificity was then determined by a melt curve analysis. Relative amounts of cDNA were analyzed with the **ΔΔCt** method.

### Western Blot Analysis

After γ-tocotrienol or PTX, or FH535 treatments, SW620 cells were scraped from culture dishes in ice cold PBS and were harvested by centrifuging. The collected cells were lysed in lysis buffer (SBS Genetech Co., Ltd). Fifty microgram of total protein was separated by 10–12% SDS–PAGE gel, and transferred onto a PVDF membrane (Millipore). The membranes were block with 5% non-fat milk in Tris-Buffered Saline Tween-20 (TBST, 1% Tween-20 in 20 mmol/L TBS, pH 7.6). The membrane was probed with specific antibodies (anti-β-actin, anti-wnt-1, anti-β-catenin, anti-cyclin D1, or anti-c-jun). The membrane was incubated with horseradish peroxidase - conjugated anti-rabbit or anti-mouse secondary antibody for 1 h at room temperature. Protein bands were visualized using a ECL kit (Santa Cruz). The protein bands were measured by FluorChem Imaging Systems (Alpha Innotech).

### Statistical Analysis

All data are expressed as means ± standard deviation (SD). The differences between the control and treated groups were assessed by one-way ANOVA test followed by Student-Newman - Keuls (SNK) test. Differences were considered significant at *P*<0.05.

### Conclusions

γ-Tocotrienol inhibited cell proliferation and induced a paraptosis-like cell death in human colon cancer cell lines both in SW620 and HCT-8. This anti-proliferative effect may at least partly involve in the suppression of the Wnt signaling pathway. Our findings suggest that γ-tocotrienol may be a potential natural reagent to prevent and treat apoptosis-resistance colon cancer.
